# Vascular cognitive impairment associated with NOTCH3 Exon 33 mutation: A case report

**DOI:** 10.1097/MD.0000000000016920

**Published:** 2019-08-23

**Authors:** Yong Sun, Yan-Jun Wei, Ying Xing

**Affiliations:** China-Japan Union Hospital of Jilin University, Changchun, China.

**Keywords:** CADASIL, NOTCH3 gene, vascular cognitive impairment

## Abstract

**Rationale:**

Vascular cognitive impairment (VCI) is a common cause of dementia. Research suggests that hereditary factors (gene mutations) play an important role in the pathogenesis of VCI, and a mutation of the NOTCH3 locus is frequently identified in affected patients. Herein, we report the case of a patient with confirmed VCI associated with a NOTCH3 exon 33 gene mutation and review the relevant VCI literature.

**Patient concerns:**

A 48-year-old man presented to our neurology clinic with gradually progressive cognitive impairment.

**Diagnoses:**

Brain magnetic resonance imaging revealed multiple punctate hyperintensities in the patient's periventricular white matter. Genetic analysis showed a c.6744C > T, p. Ala2223Val substitution in exon 33 of the NOTCH3 gene. We diagnosed thepatient with VCI secondary to a NOTCH3 gene mutation.

**Interventions:**

Donepezil (5 mg) and memantine (5 mg) daily.

**Outcomes:**

The patient showed symptom improvement at his 3-month and 6-month follow-up appointments.

**Lessons:**

This patient may have a new type of mutation that is different from the one seen in cerebral autosomal dominant arteriopathy with subcortical infarcts and leukoencephalopathy, although it involves a NOTCH3 defect. We propose that the entire NOTCH3 gene should be sequenced in patients with suspected hereditary VCI. This practice could facilitate the discovery of newpathogenic mutations and diseases.

## 1 Introduction

Vascular cognitive impairment (VCI) is a common cause of dementia,^[1]^ second only to Alzheimer's disease. The risk factors of VCI include unmodifiable characteristics (e.g., age, race, and genetic factors such as a family history of cerebral autosomal dominant arteriopathy with subcortical infarcts and leukoencephalopathy [CADISIL]) and modifiable conditions (e.g., hypertension, ischemic heart disease, diabetes, hyperlipidemia, and smoking).^[2]^ Research suggests that hereditary factors (gene mutations) play an important role in the pathogenesis of VCI, and a mutation of the NOTCH3 locus is frequently identified in affected patients. Here, we report the case of a patient with confirmed VCI associated with a NOTCH3 exon 33 gene mutation. The Ethics Committee of China–Japan Union Hospital of Jilin University approved the publication of this case report (the approval number is 2019061802). The patient provided written informed consent for all treatment and the publication of this report.

## 2 Case

A 48-year-old man presented to our hospital outpatient clinic with a 2-year history of gradually progressive memory loss of unknown etiology and a 1-year history of diminished attention and activity, depression, and loss of interest in life and his favorite pastimes. The patient's family members reported that he misidentified his relatives and could not remember the names of other people. The family stated that the patient had developed difficulty with calculations. For example, he could not correctly subtract 7 from 100. His relatives also noted that the features of the patient's illness varied in severity throughout the 2-year course.

The patient's medical history included hypertension and gout. He did not smoke or drink alcohol. He had earned a master's degree in 1996. His family history was significant for multiple cerebral lacunar infarctions in his father and cerebral hemorrhages in his mother and older sister. On physical examination, the patient's blood pressure, heart rate, and oxygen saturation were 135/91 mmHg, 75 beats/minutes, and 100% on room air, respectively, and he was normothermic. His orientation, memory, comprehension, and calculation were impaired, but his cranial nerve examination was unremarkable. The patient had normal muscle strength and tension. Deep and superficial sensory examinations showed no obvious abnormalities. His right biceps tendon reflex was enhanced, but other tendon reflexes were within normal limits. The patient had negative Babinski and positive Chaddock's signs bilaterally. His rapid alternating movement test results were poor in both hands. The patient scored 14/30 points on his Mini-Mental Status Examination (MMSE), 8/30 on the Montreal Cognitive Assessment (MoCA), and 30 on his activities of daily living (ADL) evaluation. Laboratory evaluations of his blood; thyroid, liver, and kidney function; antinuclear antibody spectrum; and vitamin B12 level showed no disorder. A rheumatoid factor study was negative.

**Figure 1:**
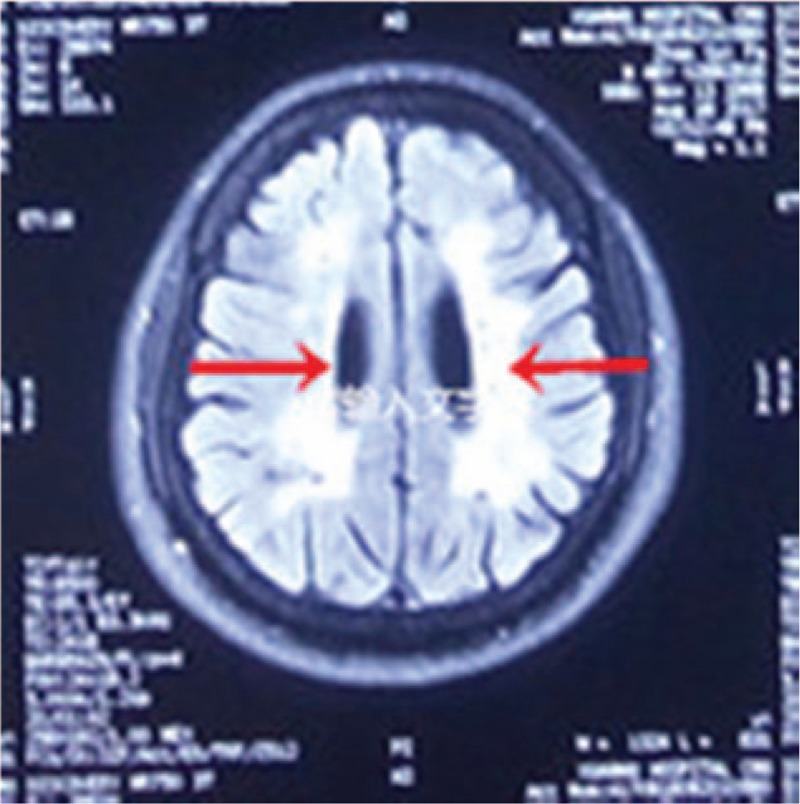
Fluid-attenuated inversion recovery imaging (August 17, 2017) shows multiple anomalous high signals in the parietal lobes and subcortical, lateral areas of the ventricles (red arrows).

**Figure 2:**
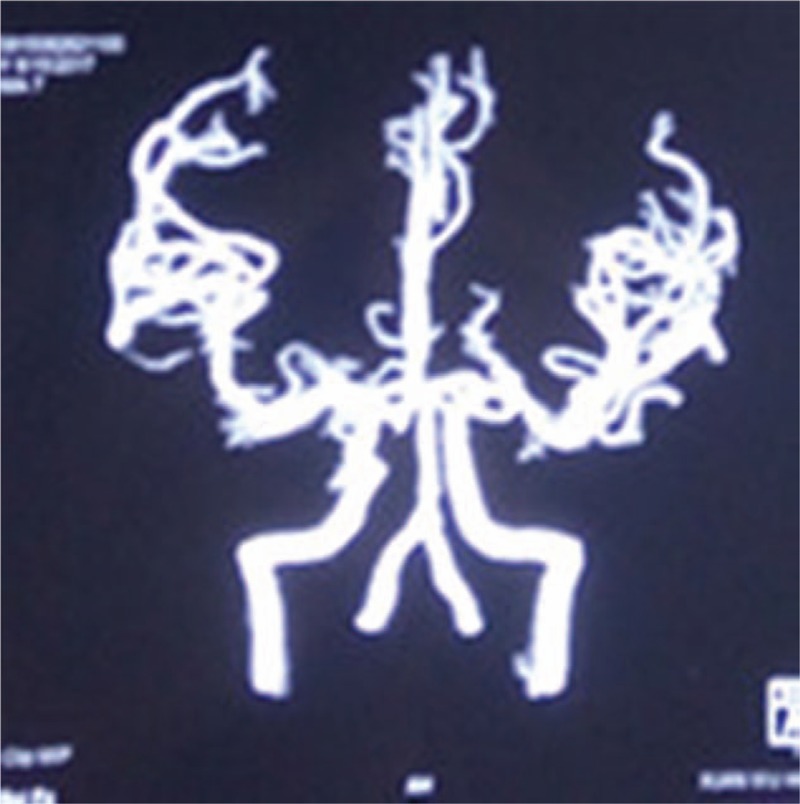
Magnetic resonance angiography (August 16, 2017) shows no abnormalities.

**Figure 3:**
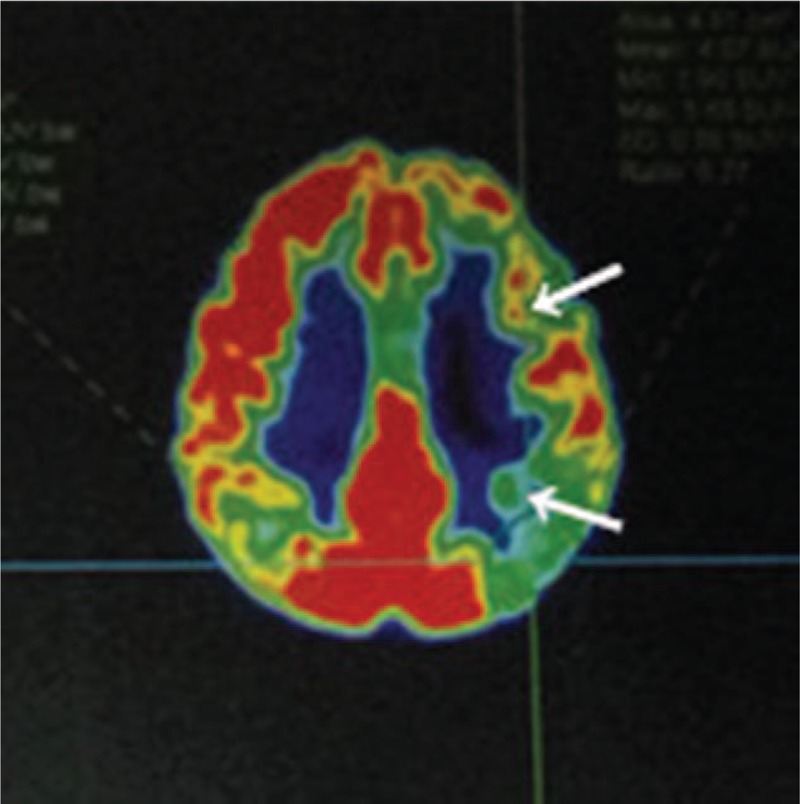
The brain metabolism image (August 18, 2017) shows decreased ^18^F-labeled fluoro-2-deoxyglucose uptake rates in the left frontal lobe, temporal lobe, thalamus, and basal ganglia compared to the rates in the corresponding contralateral areas (white arrows).

**Figure 4:**
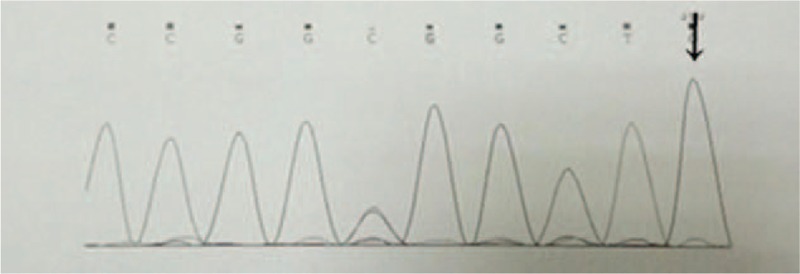
The NOTCH3 gene analysis revealed a c.6744C > T, p. Ala2223Val substitution in exon 33, but the mutation did not involve cysteine (black arrow).

Brain magnetic resonance imaging revealed multiple punctate hyperintensities in the periventricular white matter, bilateral basal ganglia, thalamus, centrum semiovale, right occipital lobe, and bilateral frontoparietal subcortical regions. These abnormal signals were markedly hypointense on T1-weighted images, hyperintense on T2-weighted images, and showed variable intensity on fluid-attenuated inversion recovery imaging (Fig. 1). Magnetic resonance angiography showed no defects (Fig. 2). Brain metabolism evaluation using ^18^F-labeled fluoro-2-deoxyglucose (^18^F-FDG) imaging showed decreased tracer uptake in the patient's left frontal and temporal lobes, thalamus, and basal ganglia compared to that in the corresponding contralateral areas, with reductions in the uptake rate of 33%, 27%, 19%, and 29%, respectively. A similar phenomenon was observed in the parietal lobes, with uptake in the left lower than that in the right (Fig. 3). Analysis of the NOTCH3 gene revealed a c.6744C > T, p. Ala2223Val substitution in exon 33, but the mutation did not involve cysteine (Fig. 4). We found no granular osmiophilic material (GOM) on immunostained skin biopsies.

Based on our findings, we diagnosed the patient with vascular dementia and prescribed donepezil (5 mg/night) and memantine (5 mg/night) to improve cognitive and other symptoms. At the 2-month follow-up, the patient scored 16/30 points on the MMSE, 10/30 on the MoCA, and 45 points on the assessment of ADL. Two months later, we increased his donepezil dose to 10 mg nightly and did not change the other treatments. Six months after beginning treatment, the patient's MMSE score was 16/30, the MoCA was 10/30, and his ADL score was 45. During the follow-up visits at 2 and 6 months, no abnormalities were found on the patient's imaging examinations.

## 3 Discussion

Hachinski and Bowler proposed the concept of VCI in 1993, describing a range of syndromes from mild cognitive impairment to dementia.^[3,4]^ According to the current classification standard, VCI can be broadly categorized as non-dementia VCI, vascular dementia, and mixed dementia.^[2]^ Both acquired and inherited subcortical small vessel disease can result in VCI.^[5]^ Genetic research shows that VCI may be caused by a gene mutation and the synergy of the resulting defect with other factors; Mutations of the apolipoprotein E (apoE) and NOTCH3 genes are frequently identified in affected patients.^[6]^ Therefore, genetic factors play an important role in the pathogenesis of VCI.

Our patient presented with cognitive impairment, hypertension, and radiographic evidence of cerebral small vessel disease (CSVD). Therefore, we considered a diagnosis of CSVD first. Further, NOTCH3 gene sequencing revealed a C.6744C > T, p. Ala2223Val substitution in exon 33, although the mutation did not involve cysteine. Thus, our patient had CVSD associated with a NOTCH3 substitution mutation – CADASIL is the most common example of this entity.^[7]^

At the time of our literature review, over 300 types of disease-causing mutations had been reported (data from The Human Gene Mutation Database). Additionally, we found many reports about NOTCH gene mutations that did not involve cysteine (Table 1). Our review indicated that pathogenic mutations occur in extracellular encoding exons 2 to 24, most frequently in exon 3 or 4.^[25,26]^ Joutel et al reported that 70% to 80% of French NOTCH3 gene mutations occur in exon 3 or 4, and exon 4 mutations are the most common.^[27]^ Markus et al reported that in the UK family of CADASIL-related NOTCH3 gene mutations, 73% are located in exon 4 and 8% in exon 3.^[28]^ German studies showed that 58.3% of mutations occur in exon 4.^[29]^ Further, similar Chinese studies showed that the NOTCH3 gene mutation is typically in exon 3 or 4, and exon 4 has the highest mutation rate.^[30]^ Although such mutations also occur in other exons,^[11,14,19–24]^ there is no previous report of an exon 33 mutation. Because we suspected CADASIL, we evaluated a skin biopsy to confirm the diagnosis. However, we found no GOM deposition in the arterioles. Thus, our patient did not meet the current Japanese diagnostic criteria for CADASIL (Table 2).^[31]^

**Table 1:**
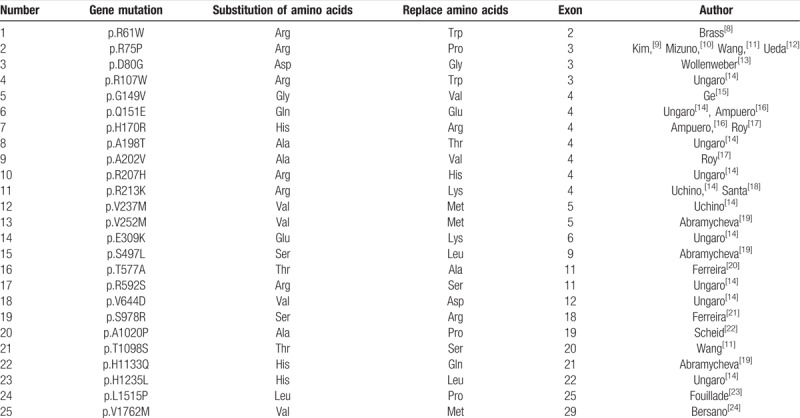
NOTCH gene mutations not involve cysteine.

**Table 2:**
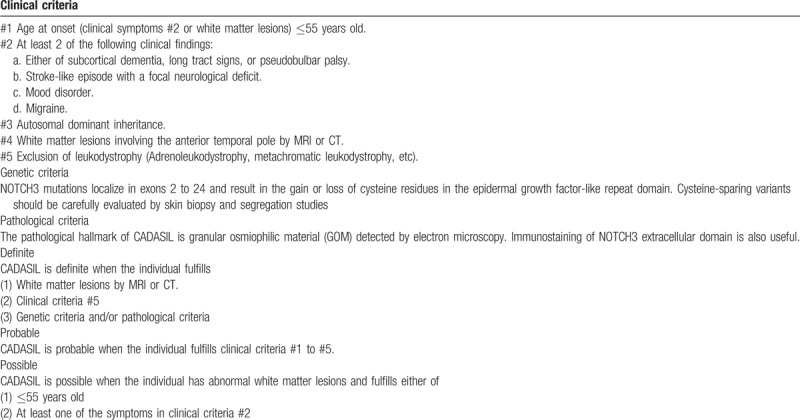
New diagnostic criteria for CADASIL in Japan.

Positron emission tomography findings typical of VCI include asymmetric uptake of ^18^F-FDG in the left and right cerebral cortices. Additionally, sugar metabolism in grape-decreased areas not only relates to the cerebral cortex but also lesions in the basal ganglia and thalamus.^[32]^ Our patient's cerebral metabolic defects and symptoms fulfilled the VCI criteria.^[33]^ Given his degree of cognitive impairment, the patient also met the criteria for the diagnosis of vascular dementia.

This patient may have a new type of mutation that is different from the one seen in CADASIL, although it involves a NOTCH3 defect. There are 2 reasons for this conclusion. Firstly, the gene mutation in our patient is significantly different than those described in previous reports involving many CADASIL mutation sites and types. Further analysis of his family's genetic map is needed to clarify whether this mutation is hereditary. Secondly, CADASIL is a systemic disease that can be diagnosed by detecting GOM deposition in skin biopsies. Although skin biopsy examination lacks sensitivity, it is highly specific for a pathological diagnosis.^[34,35]^ Our patient's skin biopsy had no GOM deposition. However, we need to confirm the accuracy of the results. Therefore, in this case, the diagnosis is VCI secondary to CADASIL resulting from an atypical NOTCH3 gene mutation or non-CADASIL-associated VCI secondary to a NOTCH3 gene mutation. Further research is needed to clarify the pathogenesis. Meanwhile, we propose that the NOTCH3 gene should be completely sequenced in patients with suspected hereditary VCI. This practice could facilitate the discovery of new pathogenic mutations and diseases.

## Author contributions

**Data curation:** Yong Sun.

**Supervision:** Ying Xing.

**Writing – original draft:** Yong Sun.

**Writing – review & editing:** Yan-Jun Wei.
